# Network-Based Method to Investigate the Promoted Cell Apoptosis Mechanisms of Oridonin in OSCC through the RNA-Transcriptome

**DOI:** 10.1155/2023/5293677

**Published:** 2023-03-16

**Authors:** Guohui Wu, Yusheng Guo, Yang Liu, Xiangsheng Cai, Tanggang Deng, Tianchu Pei, Long Huang, Kai Chen, Xuan Pan

**Affiliations:** ^1^School of Clinical Medicine, Guangdong Pharmaceutical University, Guangzhou 510000, China; ^2^Department of Stomatology, The First Affiliated Hospital of Guangdong Pharmaceutical University, Guangzhou 510000, China; ^3^Department of Neurology, Second Medical Center of Chinese People's Liberation Army General Hospital, Beijing 100089, China; ^4^Center for Medical Experiments, University of Chinese Academy of Science-Shenzhen Hospital, Shenzhen 518106, China

## Abstract

The morbidity of oral cancer is high in the world. Oridonin is a traditional Chinese medicine that can effectively inhibit oral squamous cell carcinoma (OSCC) growth, but its mechanism remains unclear. Our previous data showed that oridonin inhibited CAL-27 cell proliferation and promoted apoptosis. Herein, we explored the mechanism and target of oridonin in human OSCC through RNA sequencing and integration of multiple bioinformatics analysis strategies. Differences in gene expression can be analyzed with RNA sequencing. Kyoto Encyclopedia of Genes and Genomes (KEGG), Gene Ontology (GO), gene set enrichment analysis (GSEA), Disease Ontology (DO), and other enrichment analyses were used to evaluate differentially expressed genes (DEGs). Protein–protein interaction (PPI) networks were built via the STRING database. It was found that tumor necrosis factor (TNF) signaling pathway, cytokine–cytokine receptor interaction, and nuclear factor-kappa B (NF-kappaB) signaling pathway were associated with the therapeutic effects of oridonin in OSCC. Three key genes (BIRC3, TNFSF10, and BCL6) were found to associate with cell apoptosis in OSCC cells treated with oridonin. Quantitative PCR assays verified the expression of apoptosis-related DEGs: TNFSF10, BIRC3, AIFM2, BCL6, BCL2L2, and Bax. Western blots were employed for verifying proteins expression associated with DEGs: cleaved caspase 3, Bax, Bcl-w, anti-cIAP2, and anti-TRAIL. In conclusion, our findings reveal the molecular pathways and targets by which oridonin can treat and induce cytotoxic effects in OSCC: by affecting the signaling including TNF, NF-*κ*B, and cytokine-cytokine receptor interaction and by regulating the key gene BIRC3, TNFSF10, and BCL6. It should be noted that further clinical trial validation is very necessary. Combined with current research trends, our existing research may provide innovative research drugs for the treatment of OSCC.

## 1. Introduction

As one of the most common cancers afflicting all human beings, oral squamous cell carcinoma (OSCC) has long been a research hotspot. The morbidity of OSCC is increasing worldwide; in addition, more than 300,000 new patients are diagnosed with the disease every year; and despite some breakthroughs in diagnosis, treatment, and prognosis, the 5-year overall survival rate is only around 50% [1, 2]. The risk factors are complex, which are related to unhealthy daily habits, for example, smoking, drinking, vitamin deficiency, virus infection such as human papillomavirus and hepatitis C, working environment such as dust exposure, and family genetics [3]. OSCC is common in the lateral edge of the tongue, soft palate, and anterior floor; the typical clinical manifestations are superficial ulcer, infiltration, bleeding, and nodules [4]. Patients usually have dysphagia, earache, tongue movement limitation, cervical and submandibular lymph nodes, weight loss, and loss of sensory function, which seriously affect patients' quality of life.

Currently, the treatment for OSCC includes surgery, radiotherapy, chemotherapy, and targeted drug therapy [5]. For disease control and to manage resectable cancer, these treatment methods can be used alone or in combination, but may be accompanied by a variety of adverse effects and a high recurrence rate [6]. Therefore, better treatments for OSCC and improved prognosis are needed. It is worth mentioning that after the tongue squamous cell carcinoma CAL-27 cell line was discovered and successfully cultured in 1982, researchers used it to conduct a large number of molecular biology experiments, so whether at the cell level or at the animal level, this cell is considered to be a typical cell line with research value [7].

Oridonin, an enantiomeric kaurane diterpene, is isolated from *Rabdosia rubescens*, which is a traditional Chinese medicine with antitumor, anti-inflammatory, and antibacterial effects [8]. The antitumor activity of oridonin has been widely studied. Oridonin can effectively inhibit viability of a variety of tumor cells, mainly by inhibiting cancer cell proliferation, promoting apoptosis, but also triggering autophagy [9, 10]. Yang and others confirmed that oridonin has antilung cancer pharmacological activity through inhibition of AMPK/Akt/mTOR-dependent autophagy and reduced cisplatin resistance through activation of apoptosis signaling pathways [11].

Tumor cell apoptosis is essential to life. Apoptosis is mainly realized through the caspase family. The mitochondria contain many proapoptotic proteins, for example, apoptosis inducing factor (AIF), Smac (second mitochondria-derived activator of caspases), or DIABLO (direct IAP-binding protein with low pI); Smac/DIABLO interacts and antagonizes inhibitors of apoptosis protein (IAP), thereby activating caspases to induce apoptosis, opening up a new direction for the study of apoptosis [12, 13].

Recent finding indicated the effective inhibition of oridonin against OSCC cell proliferation through affecting cell cycle and apoptosis [14, 15]. However, the molecular pathways and targets of oridonin-induced apoptosis in OSCC cells are still unclear, and the existing reports do not use transcriptome sequencing. Here, we used high-throughput sequencing data for comprehensively analyzing the gene expression of CAL-27 cells after oridonin induction in cellular level, as well as exploring its mechanism and potential targets in cells.

## 2. Materials and Methods

### 2.1. Cell Line and Treatment

The cell line CAL-27 from Shanghai Cell Bank (Shanghai, China) was incubated with DMEM containing 10% fetal bovine serum (FBS) and 1% penicillin/streptomycin under 37°C and 5% CO_2_, which were all from Gibco (Waltham, MA, USA). 3 × 10^5^ cells per well plated in a 6-well plate were divided into the control group having complete medium containing 0.1% dimethyl sulfoxide (DMSO, Sigma, USA) and oridonin group having complete medium containing 10 *μ*mol/L oridonin (purity: 99.81%; Selleck, USA) and incubated for 48 h.

### 2.2. Cell Viability Assay

Within 96-well plates, trypsin-digested CAL-27 cells (Gibco, USA) were grown throughout 24 hours. 100 *μ*L fresh medium containing oridonin (5, 10, 15, and 20 *μ*M) was supplemented to the oridonin groups, while the control group containing 0.1% DMSO. Cells were cultured for 48 h followed by addition of 10 mg/L Cell Counting Kit-8 (Beyotime, China) with additional 1 h incubation. CCK-8 detects cell proliferation and cytotoxicity by WST-8 compounds. WST-8 is reduced to orange-yellow formazan by dehydrogenases in the mitochondria, and the lighter the color, the lower the number of cells. Subsequently, a spectrophotometer (Thermo Fisher, USA) was employed to determine the optical density at 450 nm, and the results were analyzed with statistical software.

### 2.3. Cell Apoptosis Assay

After 24 hours incubation for cells in 6-well plates, fresh medium containing oridonin (5, 10, 15, and 20 *μ*M) was added and fresh medium containing 0.1% DMSO in the control group, followed by 48 h incubation. Apoptosis and necrosis were subsequently determined utilizing annexin-V/propidium iodide (PI) double label assays (Keygen Biotechnology, China). We washed the cells and then transferred to binding buffer; after that, two stains were added for staining and placed in the dark for 20 minutes. The result was calculated using FlowJo software.

### 2.4. Transcriptome Sequencing

The cell RNA of CAL-27 was obtained with Trizol reagent (Thermo Fisher, USA) based on the manufacturer's instruction. The transcriptome of an oridonin group and a control group was sequenced in Beijing NuoheZhiyuan Technology Co., Ltd. (Beijing, China). Briefly, the mRNA containing poly A tails enriched using oligo (DT) magnetic beads was utilized for synthesizing double-stranded cDNA; then, cDNA was connected with tails using sequencing connectors following the end repair. Using AMPure XP beads, cDNA between 370 and 420 base pairs were identified with subsequent PCR amplification. The library at 1.5 ng/*μ*L obtained by purifying PCR product using AMPure XP beads was assayed for insert size via the Agilent 2100 Bioanalyzer, quantified for the valid concentration (>2 nm), and subjected for Illumina sequencing.

### 2.5. Transcriptome Analyses

To screen for DEGs with significant expression levels in the oridonin and control groups under different conditions, a standardized method was used for differential analyses, which was mainly divided into three steps. First, we normalized the original read counts using the edgeR package, and different sequencing depths were proofread and corrected. Second, the negative binomial distribution statistical model was used for hypothesis testing (*p* < 0.05). Third, following the subsequent analyses with the edgeR package, the thresholds that ∣log2 fold change | >1 and *p*adj < 0.05 were set [16, 17]. After analyzing the upregulated and downregulated genes with significant differences, to determine the effects of gene groups under different expression conditions as a whole, enrichment analyses according to the principle of hypergeometric distribution were used to annotate and classify thousands of genes, and a coexpression network pathway of DEGs was constructed. Enrichment analyses of Gene Ontology (GO), Kyoto Encyclopedia of Genes and Genomes (KEGG), and Disease Ontology (DO) were conducted via cluster Profiler software, followed up gene set enrichment analysis (GSEA) and protein–protein interaction (PPI) network construction using the STRING database. The results were validated using RNA sequencing (RNA-seq), and trend data on apoptosis-related DEGs were acquired.

### 2.6. qPCR Analysis

CAL-27 cells cultivated within 6-well plates were divided into a control group and an experimental group (10 *μ*M oridonin) and cultured for an additional 48 h. The cDNA was synthesized from a total RNA template by Evo MMLV RT premix (Accurate Biotechnology, China) and subjected to qPCR, which was performed in the qTower3g instrument (Analytical Jena AG, Germany) using an SYBR Green Kit (Accurate Biotechnology). The RNA expression was determined via 2^-*ΔΔ*CT^ method with GAPDH for date normalization.

### 2.7. Western Blot

The protein lysates of CAL-27 cells in the control and drug groups (oridonin: 0, 5, 10, and 15 *μ*M) were yielded with RIPA buffer on ice. Each set of protein samples was diluted to 5 *μ*g/*μ*L. PVDF membranes including identical amounts of total protein after electrophoresis were blocked using Quickblock^tm^ buffer (Beijing, China) and incubated with primary antibody against Bax (#2772, CST), cleaved caspase 3(#9661, CST), Bcl-w (#2724, CST), *β*-actin (#4970,CST), anti-cIAP2(ab32059, Abcam), and anti-TRAIL (ab231063, Abcam) at 4°C for 12 hours, accompanied by being incubated to secondary antibody (#98164, CST). Then, protein bands were visually analyzed using ECL Kits (Fude, China), and images were captured using a Tanon4600 camera system (Tanon, China).

### 2.8. Statistical Analyses

Minimum of three separate experiments validated all data. GraphPad and R software were used to analyze means ± standard deviations of all data. Student's *t*-test or one-way ANOVA was adopted. *p* < 0.05 was accepted as having statistical significance.

## 3. Results

### 3.1. Oridonin Inhibits CAL-27 Cell Proliferation

**Figure 1:**
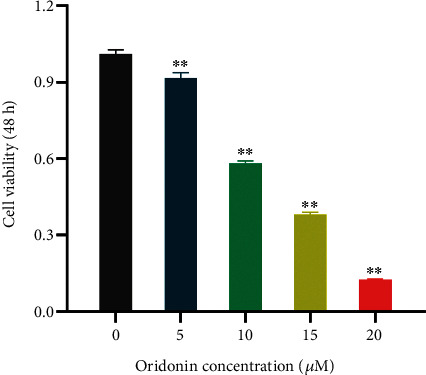
Cytotoxicity of oridonin against CAL-27 cell on the CCK-8 assay. The effect of different concentrations of oridonin on the proliferation of CAL-27 cells after 48 h. Data represent the mean ± standard deviation of three independent experiments. *p* < 0.05 is statistically significant, compared to control group: ^∗^*p* < 0.05, ^∗∗^*p* < 0.01, and ^∗∗∗^*p* < 0.001.

CAL-27 cells were treated with 5, 10, 15, and 20 *μ*M oridonin. After 48 h, cell proliferation analyses were performed. The results demonstrated that the optimal drug concentration was 10 *μ*M. Within a certain concentration range, the higher the drug concentration of oridonin, the lower the degree of proliferation of CAL-27 cells, and the two are in a linear relationship (Figure 1).

### 3.2. Oridonin Induces CAL-27 Cell Apoptosis

**Figure 2:**
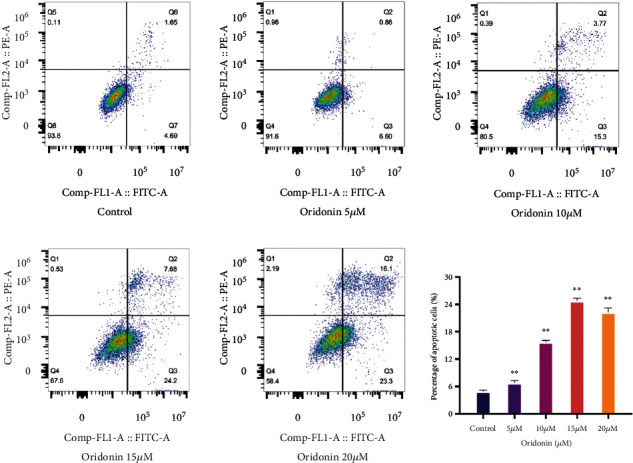
Assessment of apoptosis in CAL-27 after treatment with oridonin for 48 h using flow cytometry. The results represent mean ± SD of three independent experiments. *p* < 0.05 is statistically significant, compared to control group: ^∗^*p* < 0.05, ^∗∗^*p* < 0.01, and ^∗∗∗^*p* < 0.001.

**Figure 3:**
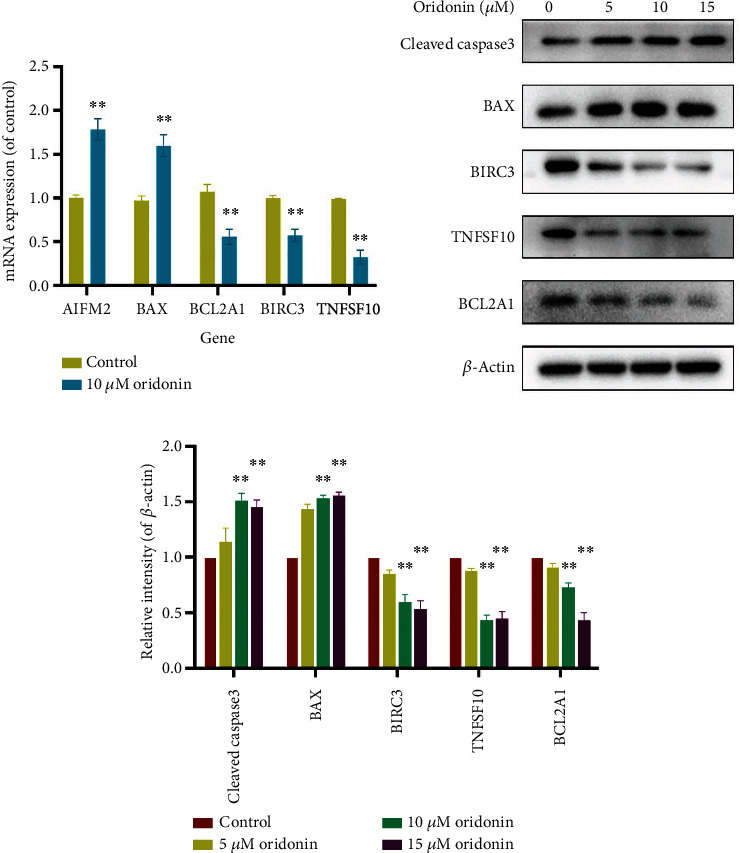
Verification of key genes. (a) mRNA expression in CAL-27 cell determined via qRT-PCR. (b, c) Protein expression in CAL-27 cell determined via Western blot. Compared to control group: ^∗^*p* < 0.05, ^∗∗^*p* < 0.01, and ^∗∗∗^*p* < 0.001.

Flow cytometry analysis showed a gradual increase in the rate of apoptosis of CAL-27 cells induced by different concentration gradients of oridonin (Figure 2). The expression of cleaved caspase 3 and Bax of CAL-27 cells significantly increased after oridonin treatment; on the contrary, antiapoptotic protein Bcl-w, anti-cIAP2, and anti-TRAIL were significantly decreased, and anti-TRAIL expression was significantly decreased at 5 *μ*M and 10 *μ*M and then slightly increased at 15 *μ*M (Figures 3(b) and 3(c)). These results indicated that oridonin can induce CAL-27 cell apoptosis dose-dependently, and apoptosis may be achieved through the mitochondrial pathway and the death receptor pathway, which depends on the cascade of caspase family.

### 3.3. Significant DEG Numbers

**Figure 4:**
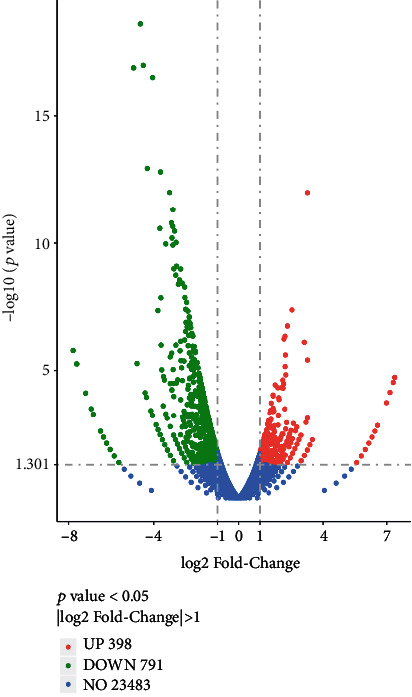
Volcano map shows the levels of DEGs in the oridonin and control groups.

The distribution of DEGs was analyzed using a volcano map. In the oridonin and control groups, there were a total of 1189 significantly different genes, of which 398 genes were significantly upregulated, represented by red dots, and 791 genes were significantly downregulated, represented by green dots; significantly different genes were screened through the edgeR software, and the threshold was edgeR *p* value < 0.05 and ∣log2FoldChange | >1.0, respectively (Figure 4).

### 3.4. Enrichment Analyses of GO Function

**Figure 5:**
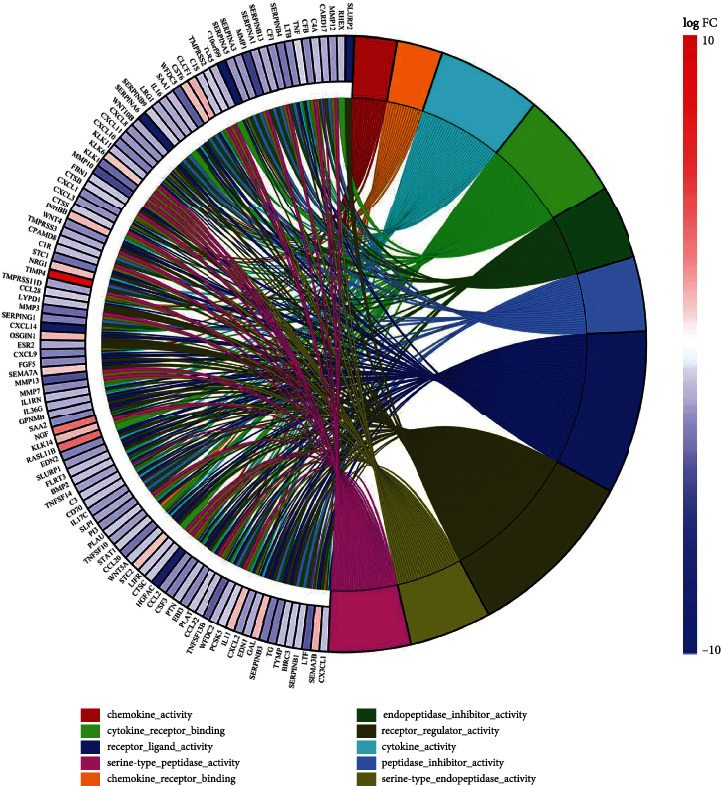
Circos plots of tightly associated GO terms and the related DEGs. Gene related to GO terms was marked with colored linking lines.

In biological process (BP), GO was mainly enriched in response to interferon gamma (IFN-*γ*), immune response activation, gene expression regulation, etc. In molecular function (MF), GO is mainly enriched in receptor ligand, receptor regulator, cytokine, chemokine activity, etc. (Figure 5).

### 3.5. KEGG Pathway Enrichment Analyses

**Figure 6:**
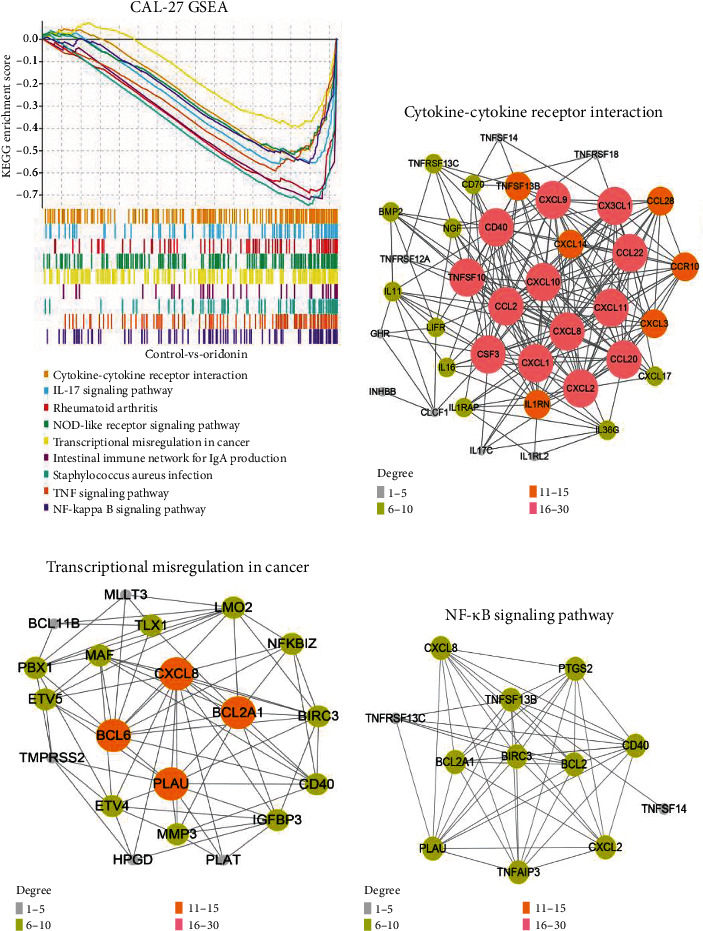
(a) Correlation network establishment. KEGG pathway dataset was utilized for identifying related pathways and key genes. (b–d) Three pathways related to oridonin were filtered.

KEGG enrichment was significant when *p*adj < 0.05. The most significant 9 KEGG pathways were selected. GSEA was mainly enriched in pathways of NF-*κ*B, cytokine-cytokine receptor interaction, TNF, etc. (Figure 6(a)), which were identified, and BCL2, BIRC3, TNFSF10, and TNFSF13B genes were identified as genes by STRING database in KEGG pathway dataset (Figures 6(b)–6(d)). These results suggest that oridonin may inhibit the proliferation and induce CAL-27 apoptosis by affecting these pathways.

### 3.6. DO Enrichment Analyses

**Figure 7:**
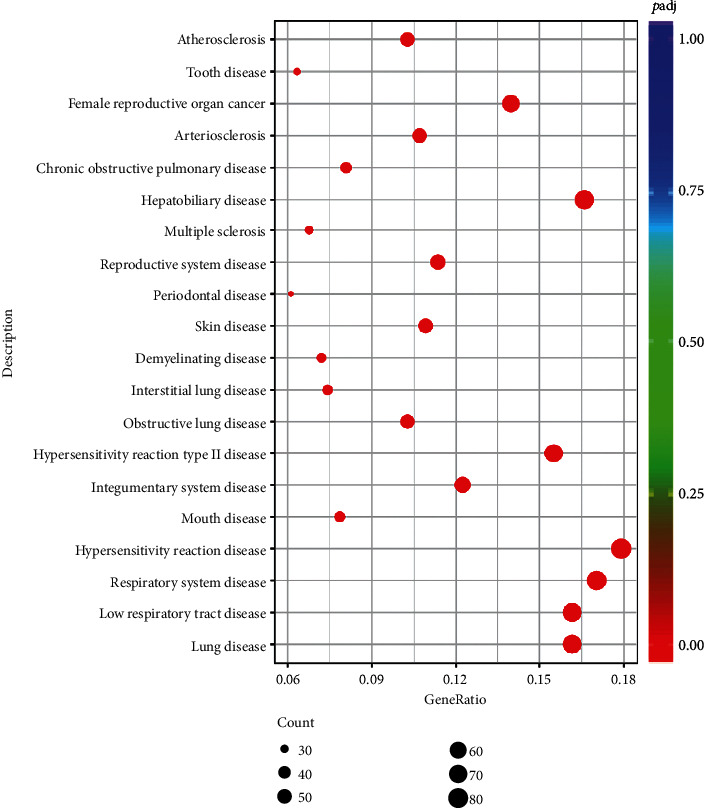
Bubble chart of DO enrichment analyses in the oridonin and control groups.

The most significant 10 terms were selected (Figure 7). DO is mainly enriched in lung disease, lower respiratory tract disease, respiratory system disease, etc. The DEGs targeted by oridonin in CAL-27 cells are closely correlated with mouth disease and periodontal disease and have biological value.

### 3.7. Identification of Key Pathways and Key Genes for Oridonin-Mediated Apoptosis

**Figure 8:**
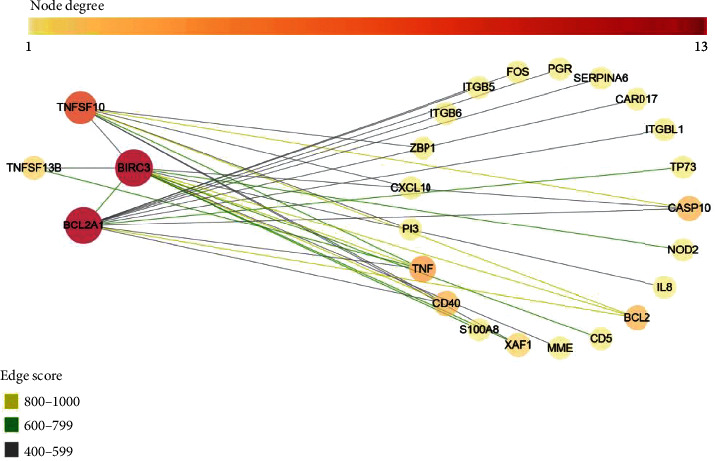
Interaction networks of differential mRNAs.

Based on the above results, the PPI network of oridonin versus the control group was analyzed. As shown in Figure 8, four central genes were selected from the PPI network, including BCL6, BIRC3, TNFSF10, and TNFSF13B. The genes associated with BCL6 expression were BIRC3, TNFSF13B, BCL2, and BCL11A. The genes associated with TNFSF10 were BIRC3, caspase 10 (CASP10), BCL2, and TNF. The main genes associated with BIRC3 were Bcl-2A1, CASP10, NOD2, BCL2, TNFSF10, TNFSF13B, and TNF. The gene associated with TNFSF13B was TNF, BIRC3, and BCL6.

### 3.8. Validation of Key Genes in Cell Apoptosis by qPCR

For characterizing the role of oridonin in critical apoptosis-related genes, we at first quantitatively determined its effects on mRNA expression of TNFSF10, BIRC3, BCL6, AIFM2, BCL2L2, and Bax. The expression levels of Bax and AIFM2 showed an upward trend in the oridonin group, but the expressions of BCL2L2, BIRC3, BCL6, and TNFSF10 showed an opposite trend (Figure 3(a)). Such results suggest that oridonin may induce apoptosis in CAL-27 cells through regulating these genes and activate mitochondrial pathway and death receptor pathway. In addition, the gene detection results were the same as the protein expression, and these apoptosis-related genes may have potential as therapeutic targets for OSCC.

## 4. Discussion

OSCC is an aggressive malignant tumor that often relapses and metastasizes, and it has high morbidity worldwide with a mortality rate of about 50%. Patients receiving standard treatment have a high recurrence rate [2]. The pathogenic factors are complex and are related to unhealthy lifestyle choices such as smoking and drinking. OSCC is currently treated with surgery, radiotherapy, chemotherapy, and targeted drug therapy. Conventional chemotherapy results in a variety of adverse reactions [5]. The antitumor activity of oridonin has been widely studied. Oridonin can effectively inhibit the proliferation of OSCC, mainly by inhibiting the cancer cell growth and cell cycle arrest, promoting apoptosis, and inducing autophagy [15]. However, there have been no reports of the effects of oridonin on the transcriptome sequence of OSCC. Thus, this research for first time determined the differences and alteration profile of mRNA expression in CAL-27 cells treated by oridonin using RNA-seq.

Enrichments of GO functions concerning mRNA expression profiles of CAL-27 cells were mainly reflected in immune response activation and responses to IFN-*γ* and type I IFN. The cell components were mainly related to the cytoskeleton, clathrin-coated endocytic vessel membrane, and luminal side of the membrane of endoplasmic reticulum, which were involved in the activities of receptor regulator and cytokine, and cytokine receptor binding, etc. Enrichment of the KEGG pathway indicated that oridonin could induce cytokine–cytokine receptor interaction in CAL-27 cells, which may participate in the anti-inflammatory and antitumor process through the signaling pathways of NF-*κ*B, TNF, IL-17, toll-like receptor, etc. The enrichment of the DO pathway is mainly reflected in respiratory system disease, mouth disease, and periodontic disease. Four central genes including BCL6, BIRC3, TNFSF10, and TNFSF13B were selected for PPI network analyses. These genes are important targets associated with each other and provide a powerful explanation for exploring the mechanism of OSCC.

In the present study, apoptosis-related genes, such as antiapoptotic genes BCL2L2, BCL6, BIRC3, TNFSF10, TNFSF13B, and apoptosis promoting gene AIFM2, were screened out to explore the mechanism and target of apoptosis. BCL2 protein family mainly dominates the intrinsic pathway of apoptosis. BCL6 was one of critical oncoproteins and therapeutic targets [18]. This study clarified that oridonin significantly downregulated BCL2L2 and BCL6 expression, which may indicate that oridonin promoted CAL-27 apoptosis by inhibiting the expression of genes BCL2L2 and BCL6.

Apoptosis-inducing factor (AIF) can induce morphological changes of cell nucleus and does not depend on caspase [19, 20]. Our study demonstrated that after oridonin stimulation, the expression of the apoptosis-promoting gene AIFM2 in cells was significantly upregulated, indicating the fundamental involvement of mitochondria in apoptosis. Inhibitor of apoptosis proteins (IAPs), promoted by the NF-*κ*B in the nucleus, promoted cell growth by reacting with mature caspase and reducing its activity [21, 22]. BIRC3 expression was significantly downregulated through oridonin treatment, which may be an important target of apoptosis.

In TNFSF10-induced signaling, the lysosome engaged in degrading BIRC2 (cIAP1)/BIRC3 (cIAP2) as well as CFLAR, requiring proteasome activity [23]. Cancer-associated fibroblasts (CAFs) have been reported to express TRAIL decoy receptors, to develop a microenvironment that promotes tumor growth [24]. Combined with our study, the expression of TNFSF10 was gradually downregulated after oridonin treatment, and the apoptosis-promoting effect was obvious at 5 *μ*M and 10 *μ*M. However, the expression was slightly increased at 15 *μ*M, possibly because TNFSF10 affects the tumor microenvironment and antagonizes death.

Oridonin has attracted more and more attention for its broad bioactivity and latent anticancer capacity. To our knowledge, RNA-seq was used for the first time to study the differences and changes in mRNA expression profile of CAL-27 cells treated by oridonin, selecting hub modules for identification of crucial pathways and genes. Eventually, pathways of cytokine–cytokine receptor interaction, TNF, and NF-*κ*B were identified; key genes were BCL6, BIRC3, and TNFSF10. This study provides a reference for better understanding the oridonin proapoptotic mechanism in CAL-27 cells, offering perspective biomarkers and targets for prevention and therapy of OSCC. In the future, we will focus on elucidating the therapeutic potential of oridonin for OSCC in animal models and clinical studies. This has important implications for the development and application of OSCC drugs.

## Data Availability

Illumina sequencing reads had been submitted to the SRA (accession number PRJNA726932), and other data available from the corresponding author upon request.

## References

[1] Meng X., Lou Q. Y., Yang W. Y. (2021). The role of non-coding RNAs in drug resistance of oral squamous cell carcinoma and therapeutic potential. *Cancer Communications*.

[2] Sasahira T., Kirita T. (2018). Hallmarks of cancer-related newly prognostic factors of oral squamous cell carcinoma. *International Journal of Molecular Sciences*.

[3] Hettmann A., Demcsak A., Decsi G. (2016). Infectious agents associated with head and neck carcinomas. *Advances in Experimental Medicine and Biology*.

[4] Zhang Y. X., Zhang B., Gao L., Xu Z. G., Tang P. Z. (2013). Clinical analysis of 318 cases of oropharyngeal squamous cell carcinoma. *Zhonghua Er Bi Yan Hou Tou Jing Wai Ke Za Zhi*.

[5] Bharadwaj R., Sahu B. P., Haloi J. (2019). Combinatorial therapeutic approach for treatment of oral squamous cell carcinoma. *Artificial Cells, Nanomedicine, and Biotechnology*.

[6] Huang S. H., O'Sullivan B. (2013). Oral cancer: current role of radiotherapy and chemotherapy. *Medicina Oral, Patologia Oral y Cirugia Bucal*.

[7] Gioanni J., Fischel J. L., Lambert J. C. (1988). Two new human tumor cell lines derived from squamous cell carcinomas of the tongue: establishment, characterization and response to cytotoxic treatment. *European Journal of Cancer & Clinical Oncology*.

[8] Ku C. M., Lin J. Y. (2013). Anti-inflammatory effects of 27 selected terpenoid compounds tested through modulating Th1/Th2 cytokine secretion profiles using murine primary splenocytes. *Food Chemistry*.

[9] Zhao X., Zhang Q., Wang Y. (2021). Oridonin induces autophagy-mediated cell death in pancreatic cancer by activating the c-Jun N-terminal kinase pathway and inhibiting phosphoinositide 3-kinase signaling. *Annals of Translational Medicine*.

[10] Che X., Zhan J., Zhao F. (2021). Oridonin promotes apoptosis and restrains the viability and migration of bladder cancer by impeding TRPM7 expression via the ERK and AKT signaling pathways. *BioMed Research International*.

[11] Yang H., Gao Y., Fan X., Liu X., Peng L., Ci X. (2019). Oridonin sensitizes cisplatin-induced apoptosis via AMPK/Akt/mTOR-dependent autophagosome accumulation in A549 cells. *Frontiers in Oncology*.

[12] Wu G., Chai J., Suber T. L. (2000). Structural basis of IAP recognition by Smac/DIABLO. *Nature*.

[13] Martinez-Ruiz G., Maldonado V., Ceballos-Cancino G., Grajeda J. P. R., Melendez-Zajgla J. (2008). Role of Smac/DIABLO in cancer progression. *Journal of Experimental & Clinical Cancer Research*.

[14] Yang J., Ren X., Zhang L., Li Y., Cheng B., Xia J. (2018). Oridonin inhibits oral cancer growth and PI3K/Akt signaling pathway. *Biomedicine & Pharmacotherapy*.

[15] Wang H., Zhu L., Feng X., Zhang H., Luo Q., Chen F. (2017). Oridonin induces G2/M cell cycle arrest and apoptosis in human oral squamous cell carcinoma. *European Journal of Pharmacology*.

[16] Anders S., Huber W. (2010). Differential expression analysis for sequence count data. *Genome Biology*.

[17] Robinson M. D., McCarthy D. J., Smyth G. K. (2010). edgeR: a Bioconductor package for differential expression analysis of digital gene expression data. *Bioinformatics*.

[18] Saito M., Novak U., Piovan E. (2009). BCL6 suppression of BCL2 via Miz1 and its disruption in diffuse large B cell lymphoma. *Proceedings of the National Academy of Sciences of the United States of America*.

[19] Susin S. A., Lorenzo H. K., Zamzami N. (1999). Molecular characterization of mitochondrial apoptosis-inducing factor. *Nature*.

[20] Cande C., Cecconi F., Dessen P., Kroemer G. (2002). Apoptosis-inducing factor (AIF): key to the conserved caspase-independent pathways of cell death?. *Journal of Cell Science*.

[21] Fulda S. (2008). Targeting inhibitor of apoptosis proteins (IAPs) for cancer therapy. *Anti-Cancer Agents in Medicinal Chemistry*.

[22] Frazzi R. (2021). BIRC3 and BIRC5: multi-faceted inhibitors in cancer. *Cell & Bioscience*.

[23] He W., Wang Q., Xu J. (2012). Attenuation of TNFSF10/TRAIL-induced apoptosis by an autophagic survival pathway involving TRAF2- and RIPK1/RIP1-mediated MAPK8/JNK activation. *Autophagy*.

[24] O'Leary L., van der Sloot A. M., Reis C. R. (2016). Decoy receptors block TRAIL sensitivity at a supracellular level: the role of stromal cells in controlling tumour TRAIL sensitivity. *Oncogene*.

